# Regional Variations in Mechanical Properties of Porcine Leptomeninges

**DOI:** 10.34133/cbsystems.0462

**Published:** 2025-12-16

**Authors:** Chenyi Lei, Wenyuan Shao, Xi Yuan, Lulu Xu, Alexander Tuzikov, Ravshan Sabirov, Semih Calamak, H. Atakan Varol, Naila Sajjad, Ijaz Gul, Peiwu Qin

**Affiliations:** ^1^ Shenzhen International Graduate School, Tsinghua University, Shenzhen 518071, China.; ^2^ Research-Engineering Center of Informational Technologies, National Academy of Sciences of Belarus, 220072 Minsk, Belarus.; ^3^ Institute of Biophysics and Biochemistry, National University of Uzbekistan, Tashkent 100174, Uzbekistan.; ^4^ Faculty of Pharmacy, Department of Basic Pharmaceutical Sciences, Hacettepe University, 06100 Ankara, Turkey.; ^5^ Institute of Smart Systems and Artificial Intelligence, Nazarbayev University, Astana 010000, Kazakhstan.; ^6^ University Institute of Biochemistry and Biotechnology (UIBB), PMAS Arid Agriculture University, Rawalpindi 46300, Pakistan.; ^7^ Department of General Surgery, Hebei Key Laboratory of Colorectal Cancer Precision Diagnosis and Treatment, The First Hospital of Hebei Medical University, Shijiazhuang 050031, China.

## Abstract

As a mechano-biological interface, the meninges dissipate external forces, maintain neuroimmune homeostasis, and dynamically regulate the brain’s microenvironment. A comprehensive study of the regional heterogeneities in meninges can improve predictions of extra-axial hemorrhage and enhance bio-fidelity of finite element (FE) modeling of head trauma under multiple injury scenarios and pathological conditions. Here, we characterized the mechanical properties of porcine leptomeninges by performing rheological shear modeling and atomic force microscopy indentation experiments. Anatomical areas encompassed the piriform, occipital, frontal, parietal, and temporal lobes, along with the cerebellum lobe. Both macromechanical and micromechanical properties indicate that the modulus of the cerebellar lobe region is much higher than that of other lobes of the pia mater. Meanwhile, the regions of the leptomeninges also displayed local mechanical anisotropy. Regional variations in the mechanical properties were further characterized by analyzing the spatial distribution in protein compositions (collagen and elastin) through 2-photon microscopy and RNA sequencing. The cerebellum lobe was found to exhibit markedly elevated levels of collagen, elastin, and cell junction proteins. Additionally, the cerebellum lobe was also identified to have markedly greater thickness compared to other lobes. Taken together, the results revealed the intricate biomechanical architecture of the leptomeninges and underscore the need to analyze its heterogeneities when modeling FE models or other computational models during traumatic brain injury.

## Introduction

**Fig. 1.**
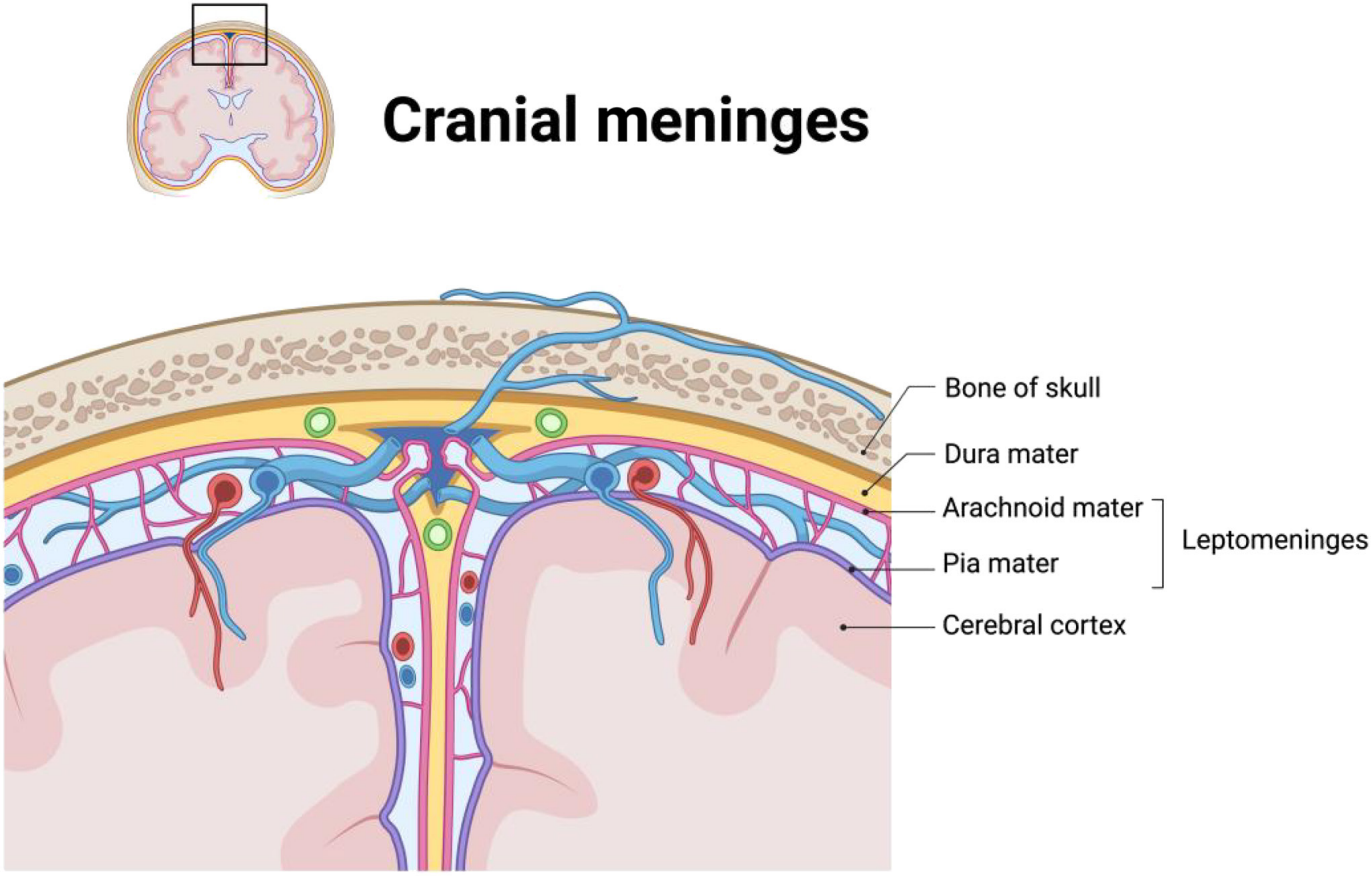
Schematic illustration of the cranial meningeal anatomy.

Despite their critical importance, the human meninges remain an underexplored tissue. Although early investigations by anatomists and neuroscientists identified the meninges as one of the fundamental brain structures [1], a comprehensive understanding of their properties and functions has not yet been achieved. The mammalian meninges are anatomically characterized as a 3-layered fibrous membrane system, covering the central nervous system (Fig. 1). The dura mater, whose name originates from the Latin for “hard mother”, constitutes the denser, superficial layer in direct contact with the skull. Within the dura lie 2 highly structured, closely associated vascularized membranes, known as the arachnoid mater and pia mater, respectively [2]; collectively, the pia–arachnoid complex (PAC) or leptomeninges. Positioned between the pia and arachnoid layers, the subarachnoid space (SAS) serves as a reservoir for cerebrospinal fluid (CSF) and accommodates both blood vessels and immune cells. Owing to tight junctions between the PAC barrier, the most well-known role of the PAC is the mechanical safeguarding of the CSF and the brain. Recent studies have demonstrated that depletion of parenchymal border macrophages (PBMs) results in the buildup of extracellular matrix (ECM) proteins and compromises the brain’s perfusion by the CSF. PBM dysfunction is linked to the aging process, and studies have demonstrated that administering macrophage colony-stimulating factor to aged mice enhances CSF dynamics [3,4]. These findings suggest that exploring the mechanical role and differences of PAC will be of great value in the immunological and physiological role of PAC.

The PAC plays a critical role as a structural damper; however, its mechanical properties are still largely unexamined. The escalating prevalence of reported traumatic brain injury (TBI) events exerts a significant impact on the world population, with annual figures indicating a minimum of 1.7 million injuries, 275,000 hospitalizations, and 50,000 mortalities. Given this context, it is imperative to investigate the mechanics of the brain–skull interface during head-onset impacts or collisions [5,6] where macroscopic loads are first transmitted through the meninges layers to cortex, followed by applied mechanical loads to parenchyma triggering a series of precisely regulated physiological responses and inflammatory reactions [7,8] that can result into an array of complex clinical symptomatology [9]. To enhance comprehension of the human head’s response to dynamic loads, various models, including finite element (FE) head models, have been developed since the 2020s [10]. These models have yielded crucial insights into the fundamental mechanisms [11,12] of TBI, particularly in contexts like road traffic accidents [13], accidental falls [14], and other impact-related incidents. The predictive accuracy of these models is highly dependent on the fidelity of the foundational data describing tissue behavior.

Considerable research efforts have been devoted to mechanically characterizing brain tissue, cranial bone, and scalp tissue [15,16]. To date, numerous studies have focused on the dura mater, because of the relative ease of isolation. Characterization of its tensile strength and other mechanical properties [17] has been conducted across multiple species, such as rat [18], human [19], and monkey [20]. Recently, these mechanical properties have been found to be associated with biomechanical properties, such as their protein content (especially as to collagen and elastin [21]). These findings carry substantial surgical and clinical implications [22]. Yet, until now, no such abundant data exist for the pia mater, arachnoid mater, or the PAC.

At the earliest time, Jin et al. analyzed the macroscopic tensile, traction, and shear properties of bovine PAC. No significant mechanical differences were observed when analyzing PAC extracted from temporal, frontal, parietal, and occipital regions [23]. Ovine PAC also showed no substantial mechanical variations between the occipital and frontal lobes [24]. These data, however, indicated a distinct mechanical response of meningeal tissue to localized loads, a point highlighted by Scott et al. [25]. The researchers showed that the inclusion of microscale and macroscale regional variability of the PAC into FE models significantly enhanced their ability to predict intracranial hemorrhages. Thus, a comprehensive understanding of how mechanical forces propagate differently across distinct anatomical structures and regions could significantly influence strategies for TBI prediction [26–28] and repair. Recently, Gloria Fabris demonstrated that while the rat’s cortical PAC was consistently thinner than its cerebellar counterpart, no significant differences in Young’s modulus (*E*) were noted across these distinct anatomical regions.

Even with evidence from anatomical data and computational models pointing to PAC heterogeneity, the systematic experimental analysis of its regional mechanical properties is notably underdeveloped. Some studies have shown that CSF is synthesized in the choroid plexus of the lateral ventricle, first passing through the cerebellum and then spreading to other regions. However, in previous reports, many studies have overlooked the mechanical properties of cerebellar regions. Previously reported animal models of PAC have been sheep or rats; however, the porcine model is the best measured model, second only to primates, due to its anatomical homology with human brain regions. The rat’s PAC stiffness is highly associated with vimentin and collagen I expression [29]. Also, studies have demonstrated the existence of localized collagen direction differences, which may lead to localized anisotropy [30]. To advance our comprehension of this intricate soft tissue, a thorough understanding of the PAC’s biochemical characteristics is crucial. The inherent heterogeneity of this inner tissue can influence strength [31], elasticity, and protective capabilities of cortex [32]. The unique biochemical and biomechanical properties of the PAC were conferred by variations in its content, organization, and interaction.

Our research focused on investigating the macromechanical and micromechanical properties of porcine PAC in different anatomical areas. We hypothesized that different anatomical structures would result in highly divergent mechanical responses, especially in the cerebellum lobe. To test this hypothesis, we first conducted oscillatory strain amplitude sweep and oscillatory frequency sweep tests in different positions of the isolated PAC to analyze the macromechanical difference. Then, atomic force microscopy (AFM) indentation–force relaxation curves and time relaxation curves were obtained from distinct PAC regions. This allowed us to analyze the influence of indentation rate, *E*, and the characteristic time relaxation constant. In addition, we characterized the tissue’s collagen and elastin difference through 2-photon microscope imaging. To verify the results, RNA sequencing (RNA-seq) was applied to pinpoint their contribution to the overall mechanical properties of the PAC tissue. This approach effectively enabled us to firstly put forward the “regional mechanical difference map” of mammalian PAC. Our results have the potential to improve the understanding of the mechanical and biomechanical function of the PAC, thereby advancing the development of FE models and informing the potential treatments of PAC-related immunity disorders and Alzheimer’s disease (AD) [33].

## Materials and Methods

### Tissue source and isolation

The experimental materials used in this study were derived from brain tissues obtained immediately after the slaughter of healthy domestic pigs. According to national regulations (Chinese Pig Slaughtering Quality Management Standards) [34], this study is exempted from review of the ethics committee. Due to the limited availability of fresh human and primate tissues, and given the anatomical similarity of brain division between humans and pigs, this study utilized 15 porcine brains (*Sus scrofa* landrace) aged (6.0 ± 1.0) months and weighed (130 ± 10) kg. The pigs were sacrificed and brains were obtained. To preserve tissue freshness, brains were harvested within 20 min following the animal’s death. Then, the blood was washed away, and the brains were stored at 4 °C. During all processing steps, to reduce the influence of proteases and RNases, the samples were continuously immersed in veterinary compound sodium chloride solution with hydrated diethylprocarbonate (DEPC) and phenylmethylsulfonyl fluoride (PMSF). RNA-seq samples were snap-frozen in liquid nitrogen and stored at −80 °C until testing. Because of obvious changes in tissue mechanics and RNA quality, all measurements were carried out within 36 h after the animal’s death.

**Fig. 2.**
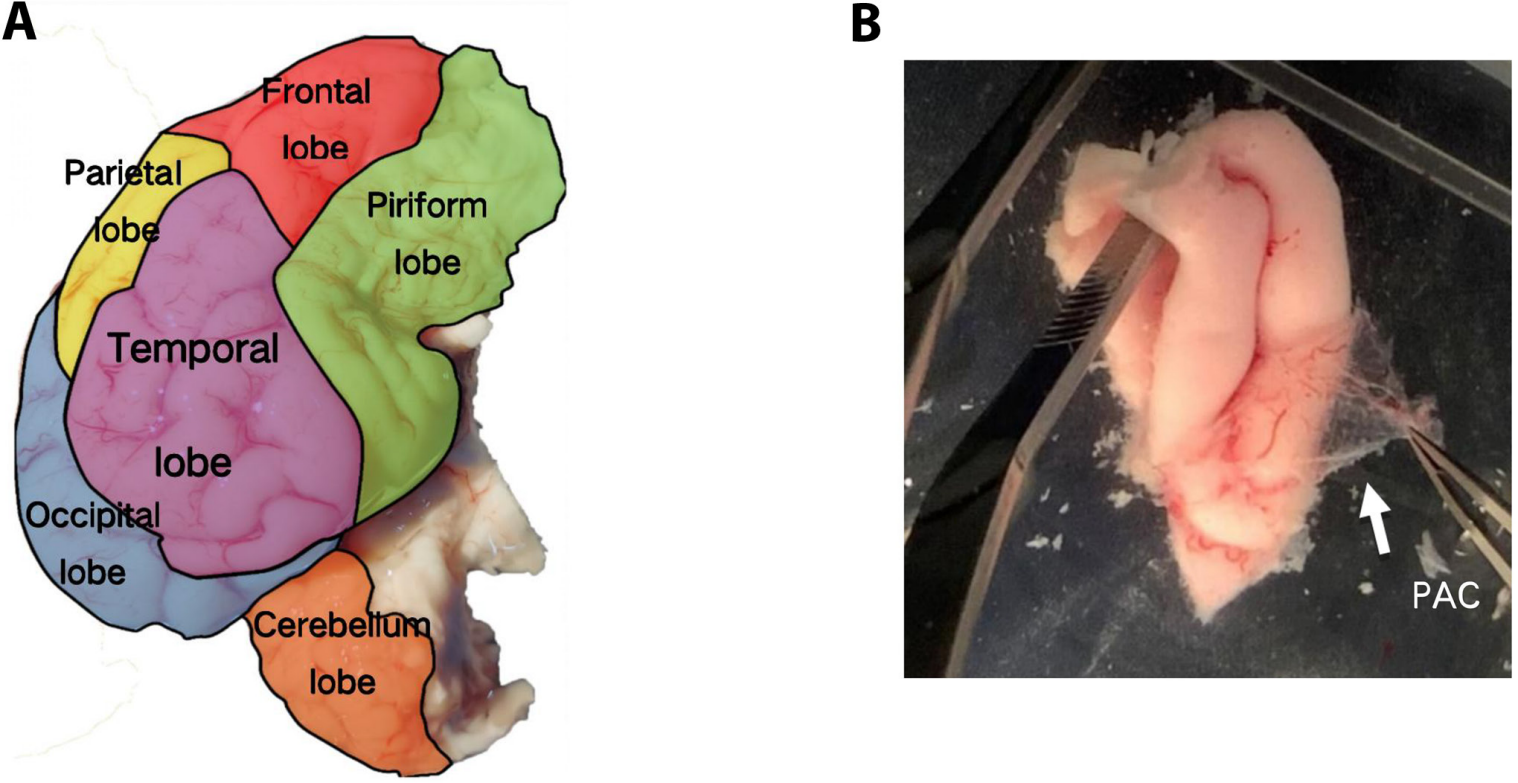
Diagram of the procine brain regions and tissue preparation. (A) Regional division of porcine meninges. (B) Stripped pig PAC in a simulated physiological environment.

In the laboratory, the dura mater was first removed, followed by the careful peeling of the PAC portions with fine-tipped, high-precision tweezers. Firstly, the regions of interest were isolated from the PAC. For all the experiments, 6 regions were tested, shown in Fig. 2A. The identification of 5 regions was based on the specific lateral cerebral lobes they enclosed: the temporal, frontal, parietal occipital, and piriform lobes [23]. The sixth region was the cerebellum, the tissue located at the posterior lower part of the brain, in the posterior fossa of the skull, and on the dorsal side of the medulla oblongata and pons. In this experiment, we only divided these 6 regions and did not distinguish between the brain tissues of boars and sows, or between the left and right brains. To minimize the impact of the adhesive layer on the tissue’s mechanical response, we immersed the tissue in the water for peeling and used a microscope cover glass to clean the adhesive layer and blood (Fig. 2B).

### Physiological oscillatory rheology

The rheological tests were performed on an Anton Paar Company instrument PTD200TA rheometer. Before starting, the removed PAC was cut into approximately 1 cm^2^ small pieces. Next, we laid the tissue on the measuring plate, with the center of the tissue facing the center of the cylinder head. Due to tissue size limitations and the stability of tissue measurements—conical heads are not recommended because PAC tends to slip—a cylindrical head with an 8-mm parallel plate geometry is applied until compacted and no further compression is possible. All measurements were performed at 37 °C, while the samples were submerged in sterile compound sodium chloride solution (DEPC and PMSF). Without soaking, rapid dehydration can be observed, affecting the experiment. To identify the most suitable oscillatory frequency sweep and oscillatory strain amplitude sweep, we performed 2 experiments. Oscillatory strain amplitude sweep measurements were conducted from 0.1% to 100% at a constant frequency of 1 rad/s. Following this, oscillatory frequency sweep measurements were taken from 1 to 100 rad/s at a fixed strain of 0.4%. For oscillatory time sweep measurements, a frequency of 1 rad/s and a strain of 0.1% to 0.3% were used, with tests repeated 3 times every 15 min [35]. The viscoelastic properties of the tissue were characterized by the storage modulus (*G*′) and the loss modulus (*G*″). The storage modulus, *G*′, represents the elastic component of the material, quantifying the energy stored during deformation. The loss modulus, *G*″, represents the viscous component, measuring the energy dissipated as heat during deformation. The ratio of these moduli, tan *δ* = *G*″/*G*′, is known as the loss tangent. This dimensionless value provides a measure of the material’s damping properties and indicates whether the viscous or elastic behavior is dominant. Each set of data was analyzed using at least 3 sets of repetitions (*n* = 3). As the data were normally distributed, we used one-way analysis of variance (ANOVA) to minimize the statistical errors in multiple group analyses.

### AFM indentation measurements and calculation

**Fig. 3.**
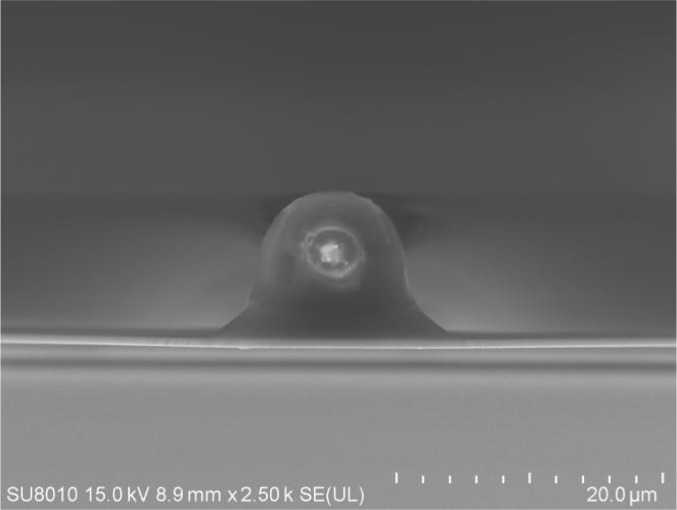
SEM image of the bead affixed under the cantilever.

To determine the stiffness of soft materials, especially soft tissues such as leptomeninges, cantilevers featuring spherical indenters are utilized. This design, characterized by a smooth spherical surface at the tip, helps to minimize indentation depth and localized pressure, providing an averaged assessment of local elasticity. Generally, there are 2 methods to get cantilevers with bead: attaching a bead with a cantilever or a commercial way from a company. Given the difficulty of attaching beads smaller than 2 μm in diameter, it is strongly recommended to purchase such small indenters as commercial cantilevers. In this experiment, we employed the method of attaching the 10-μm silica bead to the cantilever with ultraviolet (UV) glue, which is cost-efficient but has more demanding on the operator. After attachment, the position and direction of the small bead and its glue under the cantilever were then characterized using a scanning electron microscope (SEM) (Fig. 3). If it is found that the bead is not in the center or the angle or direction is incorrect, it should be discarded immediately. Each cantilever should be used starting from No. A. If the cantilever is broken or there is glue left on it, it cannot be reused. Because the test sample is the tissue of animals, and there may be a large amount of grease remaining on it, for the sake of experimental accuracy, the cantilever with bead should be replaced before each experiment.

**Fig. 4.**
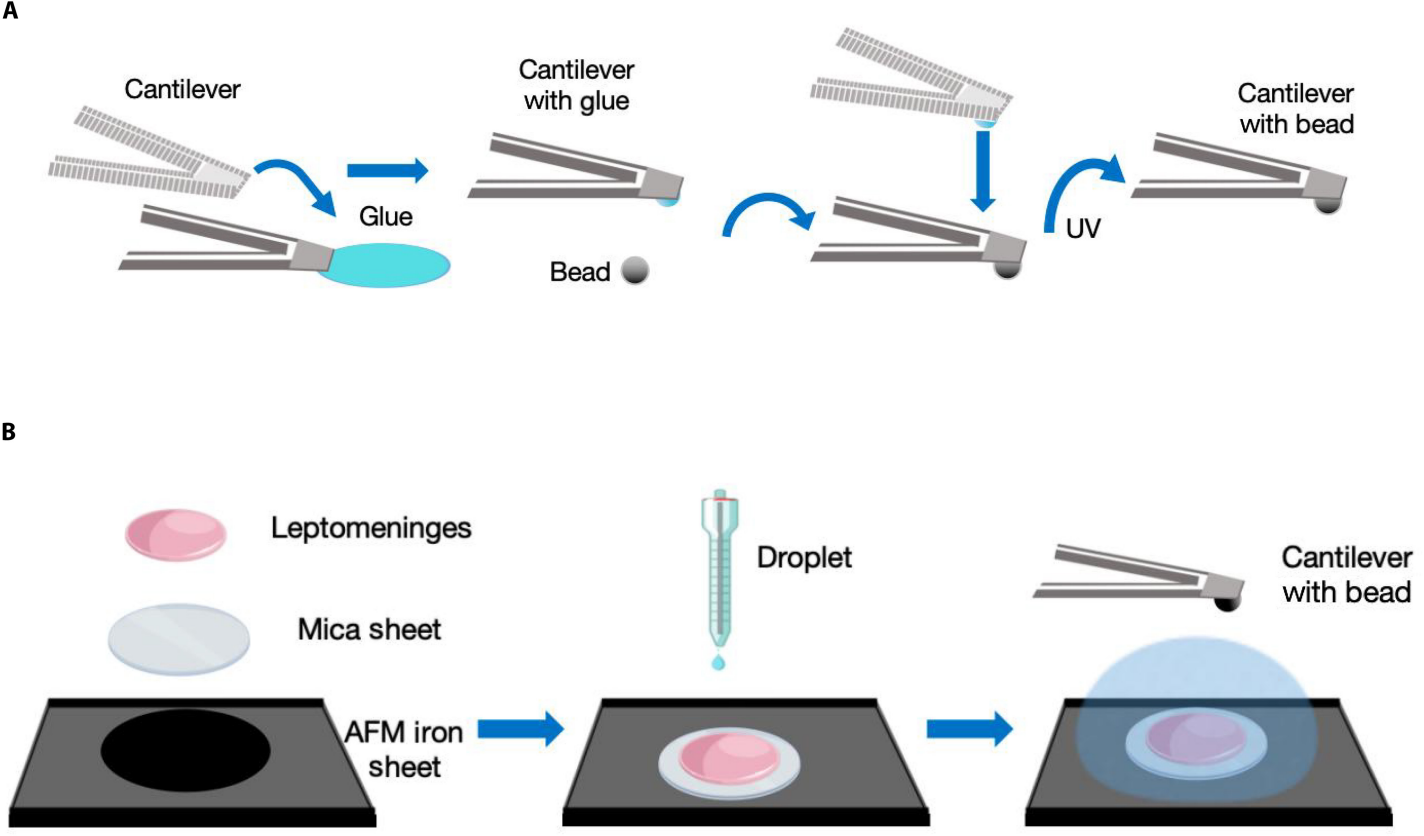
Schematic protocol of AFM indentation experiment. (A) Details on the protocol for mounting a silica bead onto a flat-tipped cantilever. (B) The procedure for securing leptomeninges on the AFM measurement plate for analysis.

The process of attachment began by selecting beads and cantilevers. In order to better maintain system stability and measurement accuracy, we utilized monodisperse 10-μm silica beads produced by Tianjin Bessler Company in China, and NPO-10 flat-head cantilevers from Bruker Company in the United States. The flat-head cantilever is mounted on the AFM probe, and the actual spring constant of each cantilever is determined by overheat calibration in the air. On one side of the glass slide, 1-μm diluted silica monodisperse beads are evenly spread, ensuring that we could see a single bead under the microscope later, and absorbent paper will be used to draw excess water from the side. On the other side of the glass slide, a thin and uniform layer of UV glue was applied [36]. The glass slide was placed under the microscope. In an AFM microscope, the area with thin and clear-edged glue was selected. Next, the edges were aligned perpendicular to the cantilever’s direction. The glue was then placed solely at the tip, not along its entire length, to avoid altering the spring constant. The cantilever was positioned so that its tip overlapped with the adhesive by only a few micrometers. The cantilever was then brought closer, and the tip of the cantilever was used to approach the edge of the glue, transferring the glue to the tip region of the cantilever. After this, the cantilever was immediately lifted, the cantilever was moved to the other side of the slide, and the tip of the cantilever was positioned against the bead. The cantilever was manually pressed down until it contacted the single bead, and the contact was kept for 20 s. Next, the cantilever was retracted from the surface. After the cantilever in the microscope had been retracted and lifted, you can see that the bead disappeared. This phenomenon could be used to evaluate whether the bead was stuck to the cantilever or not. If it is not stuck, repeat the above steps until the bead disappears after the cantilever is lifted. Then, use a UV fixer to fix it, and observe the position and amount of glue on the bead under a SEM (Fig. 4A).

Because AFM adopted an underwater mode for measurement and the PAC was not a heavy sample, it was highly likely to float underwater. For the purpose of guaranteeing the accuracy and repeatability of the experimental results, proper fixation of the sample was essential. In 2020, Shen et al. [36] embedded fresh tissues in agarose, sectioned the tissues using a vibratome, and then placed the sections on AFM-compatible dishes for tissue measurement. In 2019, Gloria Fabris floated fragments of the leptomeninges in phosphate-buffered saline and thoroughly rinsed them before being adhered to glass slides, which were previously treated with tissue and coated with a thin layer of transparent adhesive nail polish (CVS, Woonsocket, RI). To assess the influence of the adhesive layer on the mechanical behavior of the tissue, 2 control experiments were carried out. The experimental results demonstrated that the adhesive layer did not substantially alter the impact on the mechanical response of tissues [37]. Since the porcine sample is thicker than the mouse sample, and the AFM probe is highly sensitive and the downward pressure distance and force are too small [38], the fixation under the tissue does not affect the experimental results. In this experiment, in order to improve the repeatability of the experiment, we employed a pressed mica sheet from Bruker. We fixed the mica sheet to the top of the AFM’s fixed iron sheet, and then used nail polish (6046 Nail Polish, ESSIE USA) to attach the mica sheet with a size of approximately 0.5 cm by 0.5 cm tissue. After each use, the top mica sheet is removed using adhesive tape and then the next piece of tissue is pasted, thus ensuring the rapid replacement of samples and the repeatability of the experiment (Fig. 4B).

All cantilevers were individually calibrated via the thermal method (with stiffnesses between *k* = 50 and 80 kHz) before commencing each experiment. We acquired a total of 30 force maps, each typically covering an area of 5 μm × 5 μm. The grid size for each map was generally set at 5 × 5 pixels. The indentation velocities tested were in the range of *v* = 10 to 30 μm/s. For a tissue with an estimated thickness of 100 μm, the average strain rate does not affect the force map [29]. Each set of data was analyzed using at least 3 sets of repetitions (*n* = 3). The data are not normally distributed; we use the nonparametric equivalent, the Kruskal–Wallis test.

In accordance with previous research papers, the contact points of indentation curves were ascertained through the combination of the well-known algorithms. To commence, the region of the contact point was identified by means of the ratio of variances methodology as described in Ref. [39]. Once the contact point region had been determined, the double alarm technique [40] was implemented for accurate localization of the contact point. Subsequently, after calculating the linear slope in the precontact region, it was then nullified across the entire curve, thereby ensuring a more accurate and refined analysis of the indentation curve characteristics.

All data analysis was performed via custom-made Python scripts and Oxford Instrument Lgor Pro AFM Software (Version 18.10.29). By setting the AFM to contact mode, force relaxation curves were acquired; this setup held the indentation depth constant in the sample, facilitating the relaxation of the reaction force on the cantilever [41]. All measurements, including the relaxation experiments performed on the same specimens and, when possible, at the same sample positions as the indentation tests, were conducted within 24 h postmortem.

**Fig. 5.**
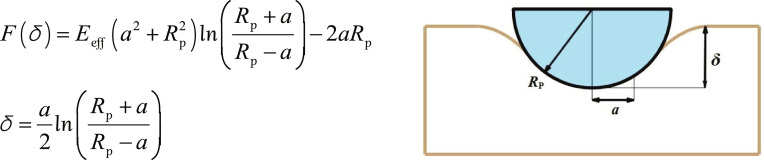
Formula specification diagram. In the equation, *ν* represents the Poisson ratio, *F* is the loading force, *δ* denotes the depth of indentation, and *E* is the Young’s modulus. The effective Young’s modulus, *E*_eff_, is defined as *E*/(1 − *ν*^2^). Additionally, *θ* is the half-opening angle of the probe, *R*_p_ is the radius of the indenting probe, and *a* is the contact radius.

The Hertz model was employed to determine the tissue’s elastic properties. However, its applicability in AFM experiments is limited to relatively small deformations, as it disregards inelastic deformation and adhesion between the probe and the sample [42]. In this experiment, the indentation depth did not reach 10% [43] of the sample thickness; thus, the Hertz model was used to fit force–time curves. The Hertz model calculates the elastic modulus from force–displacement (*f*–*d*) curves according to Hooke’s law. As the probe indents the specimen, both the applied force and the contact area increase. Consequently, the function *F*(*δ*) is determined by various parameters *ν*, *F*, *δ*, *E*, and *a* [44] (Fig. 5).

The dwell curves were fitted using a 2-term linear viscoelastic model [45]. which consists of 2 Maxwell elements in parallel with a spring (i.e., a variation of the Zener model). This corresponds to a biexponential decay of the force with respect to time:

$$F\left(t\right)=a_{0}+a_{1}\text{exp}\left(\frac{-\left(t-t_{0}\right)}{\tau_{1}}\right)+a_{2}\text{exp}\left(\frac{-\left(t-t_{0}\right)}{\tau_{2}}\right)$$ (1)

where *τ*_1_ = *η*_1_/*E*_1_ and *τ*_2_ = *η*_2_/*E*_2_ represent the time relaxation constant characteristic of the tissue, *E*_1_ and *E*_2_ are the compressive moduli and *η*_1_ and *η*_2_ are the compressive viscosities of the first and second Maxwell element, respectively [8]. The coefficient of determination *R*^2^ value was calculated for each fit, and curves with *R*^2^ < 0.985 were discarded.

### Two-photon microscopy image acquisition and analysis

For 2-photon imaging, we used custom-made non-descanned detector (NDD) instruments. The device was employed with 2 channel simultaneous acquisition via 2 NDDs and an excitation laser (with an output power range of 0 to 3 W and a tunable wavelength range of 690 to 1,300 nm). The collagen structure was imaged using second harmonic generation (SHG) signaling, and the elastin structure was imaged via autofluorescence (AF) [46]. Both images were acquired on an Olympus imaging and analysis system that was attached to an Olympus Single-Line Two-Photon Upright Microscope (FVMPE-RS) equipped with a 25× (numerical aperture 1.05) water immersion objective. We continuously adjusted the excitation wavelength to find the optimal excitation wavelength of 860 nm. Subsequently, the unfixed samples that were covered with a microscope cover glass and had water droplets on top were exposed to polarized laser light at the best wavelength of 860 nm. Emitted light was separated using 2 filter sets (RNDD1, 495 to 540 nm; RNDD2, 410 to 460 nm). Images of the *x*–*y* planes measuring 512 μm × 512 μm with a resolution of 4 μm/pixel were captured using the Olympus FVMPE-RS Software in at least 3 locations (*n* = 3) on each PAC.

A ranged threshold was set for the SHG signal to quantify collagen fibers. This threshold was maintained for all images across all conditions, and the area of regions covered by the threshold range of 15 to 35 was calculated (using ImageJ, Image Processing and Analysis in Java). The data on the diameters of collagen fibers were visualized and analyzed using ImageJ and MATLAB (produced by MathWorks) [47]. Through AF, we observed an elastin structure and calculated its content using MATLAB. We set up a threshold range from 15 to 60 and calculated the mean fluorescence intensity.

### RNA extraction and sequencing

For RNA extraction from the PAC tissue, the meninges were collected as detailed in the “Tissue source and isolation” section and stored at −80 °C until further processing. Two to three PAC samples from specific regions were combined to form a single biological replicate for each experimental group. A total of 24 samples were analyzed, with 4 biological replicates. Each set of data was analyzed using at least 3 sets of repetitions (*n* = 4) per experimental group. After thawing on ice, RNA extraction commenced with the addition of 10 silica beads to each tube, followed by 30 s of tissue homogenization using a mini bead beater. Post-homogenization, samples were centrifuged at 12,000×*g* for 10 min at 4 °C. The supernatant was then transferred to a new tube and incubated at room temperature for 5 min. Subsequently, 0.1 ml of chloroform was added, and the mixture was vortexed, incubated for 2 min at room temperature, and recentrifuged at 12,000×*g* for 15 min at 4 °C. The upper aqueous phase was transferred to a fresh Eppendorf tube, and RNA was isolated using the RNeasy Micro Kit (74004, Qiagen). RNA quality and concentration were assessed via a microplate reader. Isolated RNA was stored at −80 °C until sequencing [48]. For this, total RNA samples were sent to Beijing Tsingke Biotec for library construction and paired-end sequencing. Raw read counts were normalized to transcripts per million to account for differences in both sequencing depth and gene length, allowing for accurate comparison of gene expression across samples. Finally, graphs for biomedical data analysis and visualization were generated using the “ggpubr” (v0.4.0) and “ggplot2” (v3.4.2) [49] packages within R software (v.4.2.2). The Benjamini–Hochberg procedure was used to adjust the *P* values, and an false discovery rate (FDR) threshold of <0.05 was considered statistically significant for identifying enriched Gene Ontology (GO) terms. GO analysis is a widely used bioinformatic method to interpret large gene lists. The analysis employs statistical tests to identify GO terms that are significantly overrepresented in a set of differentially expressed genes compared to a background reference set. The resulting *P* value indicates the statistical significance of its enrichment, with lower values suggesting a nonrandom association.

## Results

### Macromechanical heterogeneity of PAC

**Fig. 6.**
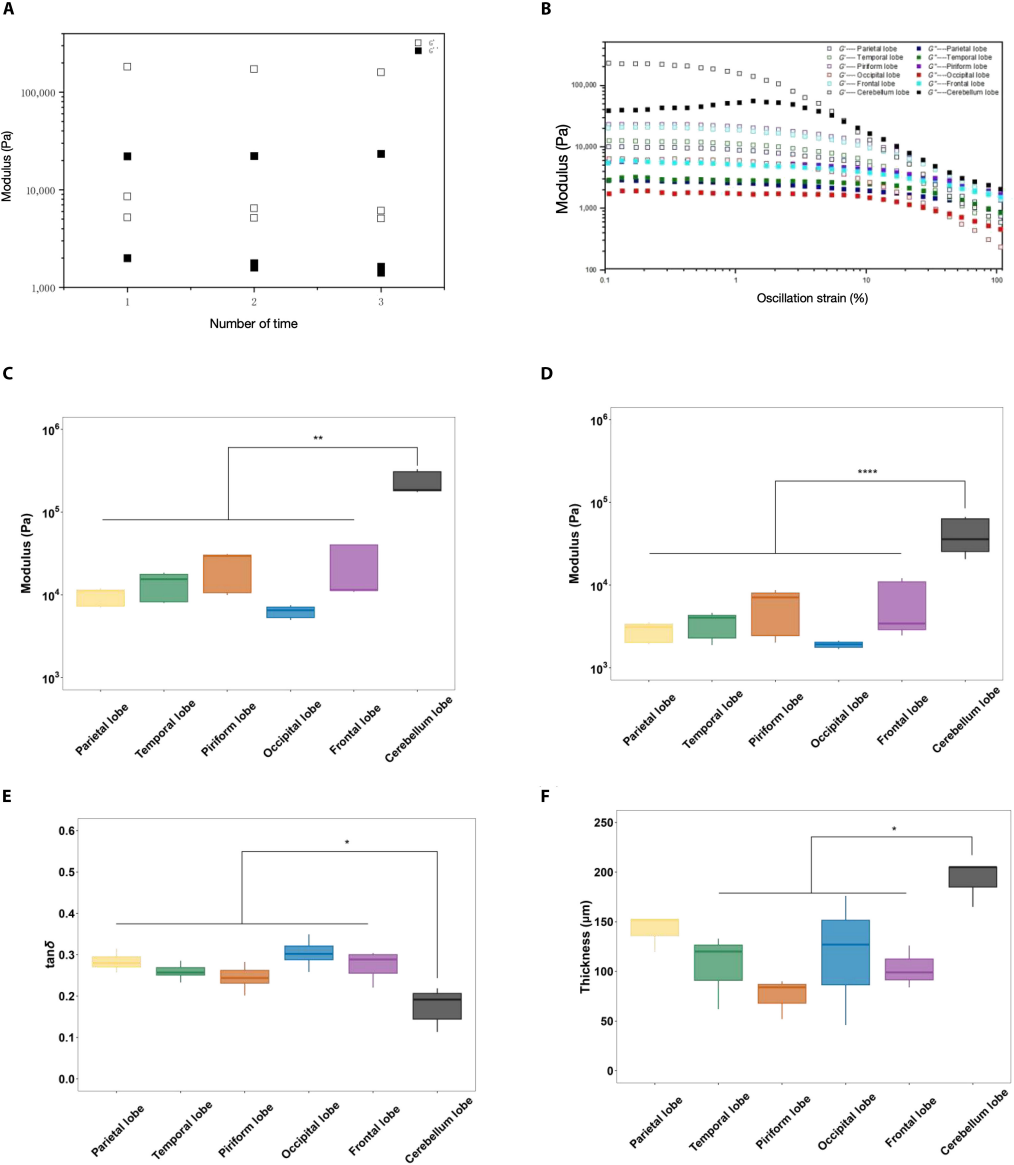
Schematic of rheological property of PAC. (A) Oscillatory time sweeps (0.1% to 0.3% strain, 1 rad/s) every 15 min for 3 times. (B) The amplitude sweep measurements at a constant frequency of 1 rad/s, from 0.1% to 100%. (C) Average value of *G*′. (D) Average value of *G*″. (E) Average value of the loss coefficient tan*δ* = *G*′/*G*″ in a frequency of 1 rad/s, from 0.1% to 0.3% strain. (F) Minimum distance between cone head and plate. All the tests were conducted at 37 °C. *P < 0.05, **P < 0.01, ***P < 0.001, ****P < 0.0001 (*n* = 3).

Throughout all experiments, all tissues were immersed in the buffer (as mentioned above in the “Tissue source and isolation” section) and placed on ice. When we used it, we take it out and put it on a plate at 37 °C for measurement. The tissue was kept moist throughout the process. Oscillatory time sweeps (0.1% to 0.3% strain, 1 rad/s, 37 °C) were performed every 15 min for 3 times (Fig. 6A). Following the initial shear, the tissue recovered immediately (no increase or decrease in *G*′ or *G*″ was observed). The measurement results indicated that neither dehydration nor degradation occurred during this period, which was different from what was previously reported that such phenomena would occur within 1 h [50,51].

The results of the amplitude sweep measurements performed at a constant frequency of 1 rad/s, ranging from 0.1% to 100%, are shown in Fig. 6B. We repeated each group 3 times and calculated the average value. A distinct increase of *G* in the cerebellar lobe region could be observed. Under the conditions of a constant frequency of 1 rad/s and a constant strain of 0.1% to 0.3%, the 2 lines representing the storage modulus and the loss modulus parallel to each other are almost constant, indicating that the material is within the elastic limit. In order to better illustrate this change, the average values of *G*′ and *G*″, as well as the loss coefficient tan*δ*, were obtained at a frequency of 1 rad/s, from 0.1% to 0.3% strain (Fig. 6C to E). The results showed a significant increase in *G*′ and *G*″ in the cerebellar region. There was no significant difference among other regions except the cerebellum.

Next, we measured the distance between the 8-mm parallel lamina and the plate on which the tissue was placed when the lamina was pressed down to the very bottom. This result could indirectly reflect the thickness of the tissue (Fig. 6F). It has been reported that the cortical rat PAC appeared thinner than that of the cerebellum during isolation and handling. Here, consistent with the previous report, the thickness of the cerebellar region was significantly higher than that of the other regions in porcine leptomeninges.

### Micromechanical heterogeneity of PAC

**Fig. 7.**
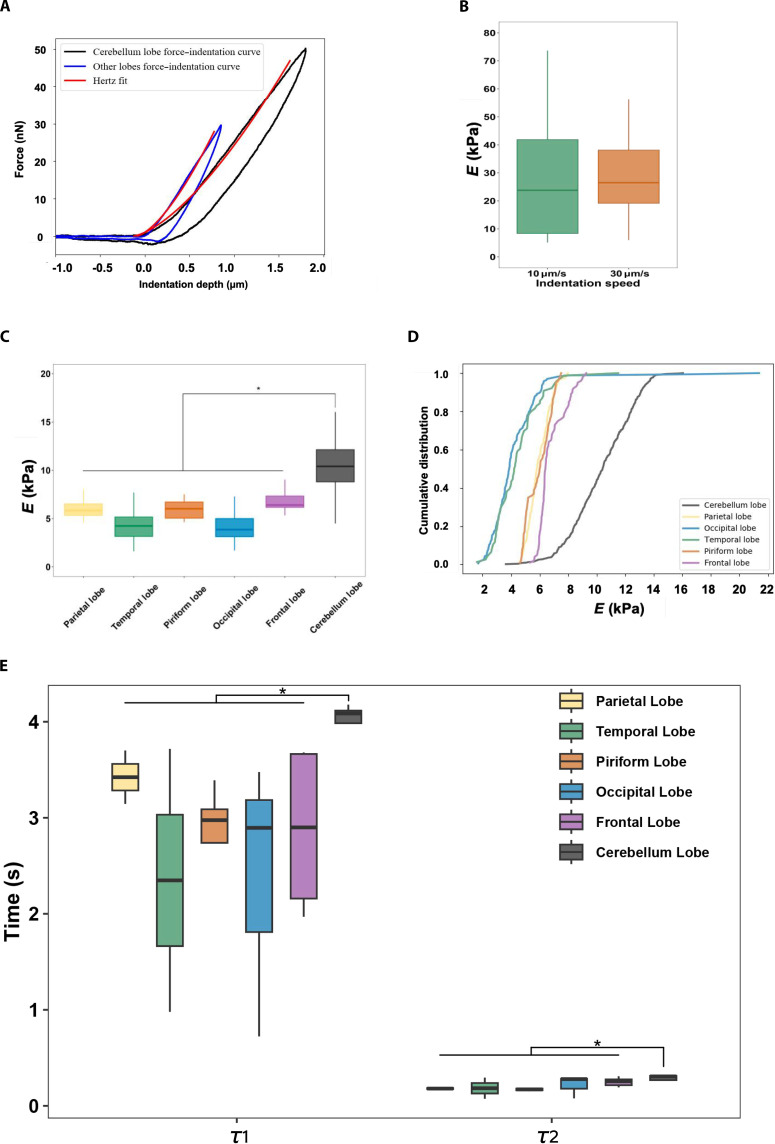
Schematic of AFM indentation experiments. (A) Example of AFM indentation curve. (B) Distribution of instantaneous Young’s modulus for near blood vessel at different indenter velocities. (C) Box plot of the distributions of instantaneous Young’s moduli for different PAC regions. (D) Cumulative distributions of Young’s moduli for different PAC regions. (E) Box plot of the distributions of time relaxation constants *τ*_1_ and *τ*_2_ for different PAC regions. *P < 0.05, **P < 0.01, ***P < 0.001.

Since the leptomeninges are soft tissues and typical biological materials, we observed a certain degree of hysteresis curves (Fig. 7A). Previous researches have reported that the indentation cantilever velocities do not affect the results. Fabris et al. tested the PAC of rats at velocities of 10 and 50 μm/s. The results showed that there was no statistical difference between the indentation cantilever velocities and Young’s modulus [29]. Later, Shen et al. [36] also found that different sampling frequencies on the same human tissue area did not affect the stiffness of the tissue. To verify the accuracy of the system again, indentation experiments were conducted at different cantilever velocities (*v* = 10 μm/s and *v* = 30 μm/s, corresponding to strain rates of about 10 and 30 s^−1^ assuming an average indentation of 1 μm). The force–displacement cycles overlapped, with a slight tendency for the material to stiffen at higher rates, in line with expectations. We tested the same area, and the results showed that for all the data of the PAC near the blood vessels, the distribution averages of Young’s modulus were around *E* = 28 kPa for *v* = 10 μm/s and *v* = 30 μm/s indentations (errors are SEM). However, no statistically significant difference was observed (Fig. 7B). All these findings show that the rates experimentally accessible by AFM are not strong enough to offer an adequate differential analysis of the tissue stiffness. Therefore, for the rest of the experiments, we decided to focus on a single indentation velocity (*v* = 10 μm/s).

After fitting the Hertz model to the first portion of the indentation domain in the curves, the instantaneous Young’s moduli *E* of different tissues were obtained for each position on the force map. Every tissue stiffness map probed at the microscale revealed significant local heterogeneities. Because the hardness of the vascular area is notably larger, and its height is significantly greater than that of other areas, and it is not uniform, this is not conducive to measure the force map and is likely to break the probe. Through several indentations, we can obviously find that the stiffness of the blood vessel area is much higher than that of other areas, and the stiffness near the blood vessel area is also higher than that of other areas. To further investigate the heterogeneity of the PAC across different brain regions, we classified all our samples into temporal lobe, frontal lobe, parietal lobe, occipital lobe, piriform lobe, and cerebellar lobe. The Hertz model fitting analysis revealed an apparent distribution of the mean values of Young’s modulus: around *E* = 10 kPa for the cerebellum lobe, and around *E* = 5 kPa for other lobes (Fig. 7C and D, *P* ≤ 0.05). Apparently, the stiffness in the cerebellar lobe region is much higher than that in the other lobes. At the scale analyzed, however, AFM indentation experiments showed that there were no significant differences among temporal, frontal, parietal, occipital, and piriform lobes in the instantaneous Young’s modulus.

Analysis of the force relaxation curves performed at velocity *v* = 10 μm/s allows us to examine the characteristic time relaxation constants of the PAC tissues. Due to the repetitive buckling during the indentation of capillaries, relaxation results were not observed for the vascularized tissues. This situation was mainly caused by the increase in external pressure, because of the spherical indenter acting on the pressurized capillaries [52]. Therefore, we only conducted force map acquisition of nonvascular areas and areas far from blood vessels. *τ*_1_ = 4.02 s and *τ*_2_ = 0.27 s for cerebellum lobe, and *τ*_1_ = 2.87 s and *τ*_2_ = 0.20 s for other lobes (Fig. 7E, *P* ≤ 0.05, errors are SEM). Substantial differences were observed across cerebellar lobe and other lobe regions of the PAC. There were no significant differences in the temporal, frontal, parietal, occipital, and piriform lobes. Then, we averaged the *E* values of each different region as we sought to observe variations in the stiffness of the whole tissue among different regions. Given that the cumulative distributions of these regions’ data were not normally distributed (as shown in Fig. 7D), a 2-sample Kolmogorov–Smirnov test was subsequently performed. This analytical approach necessitates that the PAC data under examination are statistically independent. However, because force maps encompass very small areas, this assumption is typically invalid. To facilitate meaningful comparisons, we therefore defined the PAC tissue’s test unit as the approximate size of a single arachnoid membrane from AFM. Individual unit *E* values, derived from the fitting curves, were then averaged and included in the sample group for the Kolmogorov–Smirnov test. Since our hypothesis was that we might observe variations in the stiffness of the whole tissue among different regions, we averaged the *E* values of each different region. The results of the analysis are consistent with previous studies.

### Protein expression heterogeneity of PAC

**Fig. 8.**
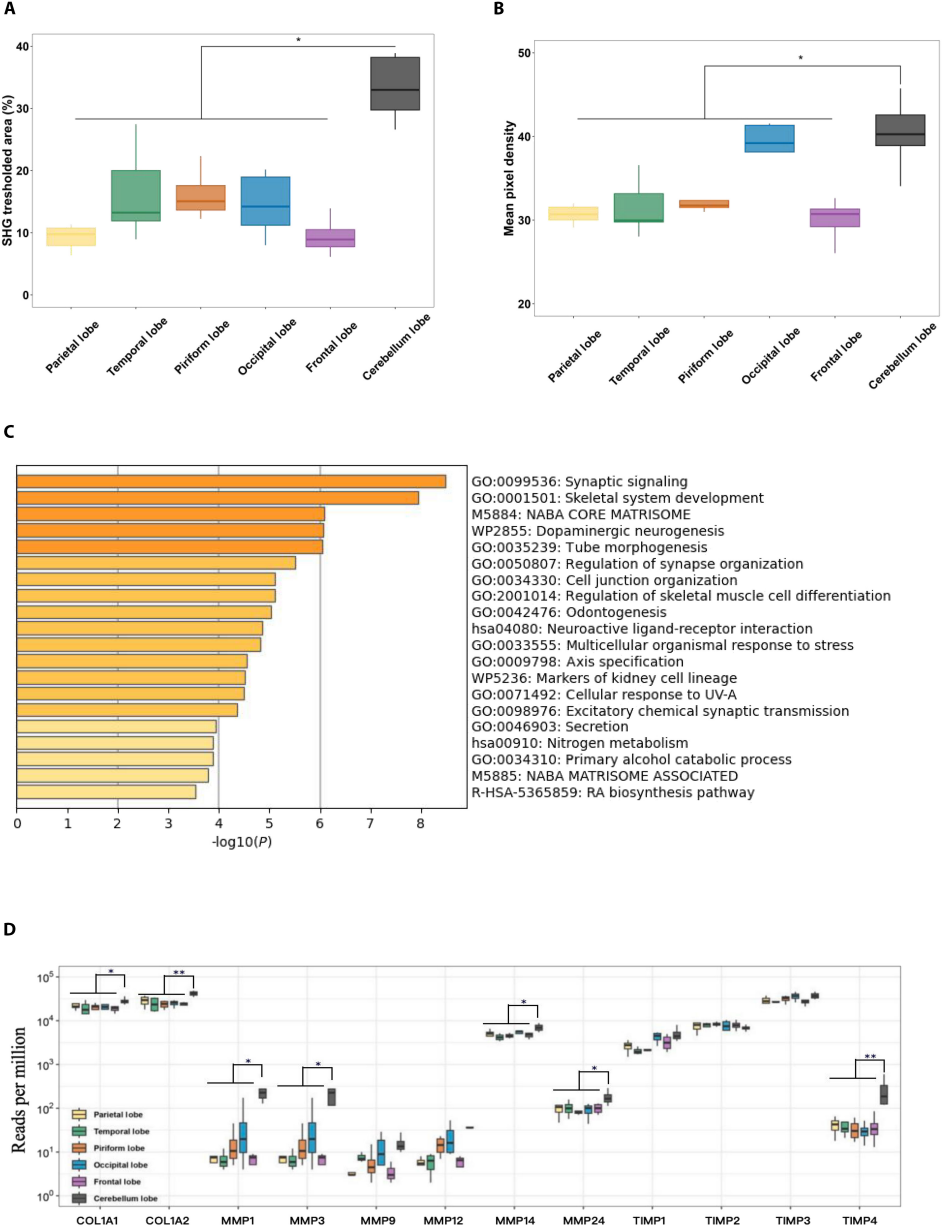
Schematic of protein expression heterogeneity of PAC. (A) Regional variations in the SHG thresholded area of the PAC, reflecting collagen content distribution. (B) Variations in mean pixel density, representing elastin contents. (C) Bar chart showing GO term molecular functions enriched by the repressed genes unique to the cerebellum lobe compared to other lobe regions. (D) Up-regulated expression of COL1 (including COL1A1 and COL1A2), membrane-bound MMPs (MMP1, 3, 9, 12, 14, and 24) and tissue inhibitor of metalloproteinases (TIMP1, 2, 3, and 4) in the cerebellum lobe, compared to other lobe regions. *P < 0.05, **P < 0.01, ***P < 0.001.

Significant regional biochemical variations exist in the porcine PAC. Prior works showed that in the mature meninges of mammals, thin cellular pillars of arachnoid trabecular cells, which contain collagen fibrils, span the SAS between the layers of the PAC [53,54]. Moreover, the collagen I staining revealed the vascular structures in rat tissues, and the vimentin staining revealed the trabecular architecture. These are 2 main factors associated with the stiffness of the PAC [29]. It is very difficult to test such a thin layer of tissue, due to its inherent geometric and anatomical heterogeneities. When the PAC is isolated from the brain, the hydrostatic pressure caused by CSF flow drops immediately, the tissue collapses, and the arachnoid trabeculae become hard to observe. Since both pigs and mice are mammals, the microstructure of their PAC is consistent with that of mice and primates. Building on these studies, the characterization of the collagen and elastin was performed. To compare the protein levels by region, it was found that the contents of collagen and elastin, positively correlated with SHG/AF signals, varied across different regions of the PAC, with significantly higher levels in the cerebellar region (Fig. 8A and B). We also found that the content of elastin in the occipital lobe was higher than that in other regions, but lower than in the cerebellar region. There was no significant difference in the contents of collagen and elastin among the other regions. This finding indirectly suggests the reason why the stiffness of the cerebellar area is greater than that of other areas. The regional variations in biochemical properties may have an important impact on the functions of different regions [21].

To further illustrate the differences in biochemical properties, we performed an RNA-seq experiment. By using GO molecular function terms, we found that many of genes in the cerebellar lobe region were significantly up-regulated, compared to other lobe regions. These genes are involved in skeletal system development, tube morphogenesis, cell junction organization, regulation of skeletal muscle cell differentiation, and secretion processes (Fig. 8C). Among the collagen-related gene sets, COL1A1 and COL1A2 showed a significant increase in the PAC of the cerebellar lobe. The expressions of the tissue inhibitor of metalloproteinases (TIMPs) and the matrix metalloproteinases (MMPs), which are membrane-bound types, were also markedly up-regulated in the PAC of the cerebellar lobe (Fig. 8D). Taken together, our observations imply that the cerebellar lobe PAC leads to an increase in collagen mediated by MMPs, the induction of TIMP expression, and the upsurge in collagen synthesis.

Our results suggest that in the cerebellar lobe region, there is an increase in collagen I, elastin association, and other junction proteins, which contributes to a higher stiffness compared to other parts of the meninges. Previously, the arrangement of collagen fibers in arachnoid layer seemed to be random [55]. Hamann et al. [56] identified the regions in the dura meninges where there were highly aligned collagen fibers, which resulted in the mechanical anisotropy of these regions via small-angle light scattering (SALS) analysis. These PAC components form an intricate network where individuals and complexes confer distinct mechanical properties. Therefore, in future research, more in-depth studies on the alignment of collagen fibers and the protein-level validation of the meninges should be taken into consideration.

## Discussion

The paucity of literature concerning the mechanical and biochemical properties of the PCA can be attributed to the technical challenges of obtaining fresh tissue samples and the difficulty in testing and isolating such a thin, soft tissue layer [57–59]. Upon isolation of the PAC from the body, the immediate drop in hydrostatic pressure from CSF flow caused the tissue to collapse, making the arachnoid trabeculae, pia, and arachnoid layers difficult to discern. Consequently, investigating the regional mechanical heterogeneity of this unique tissue becomes critically important for various computational simulations, including finite element (FE) models. For example, the FE model is essential for accurately simulating tissue response and strain propagation upon scenarios like car accidents, trauma, and brain injuries. Yet, until now, the FE model still significantly suffers from the lack of accurate experimental material characterizing regional difference in the brain–skull interface. Concurrently, measuring intracranial pressure (ICP) is a crucial aspect of clinical care for brains affected by injury or disease. Current ICP measurements are all indirect, generally relying on techniques such as ventricular fluid pressure monitoring [60], cerebral blood flow velocity, and waveform analysis with diastolic flow velocity [61], as well as pupil diffusion data [62]. Due to the compression of the meninges, or an uneven distribution of CSF, subdural and epidural monitors yield less accurate readings and are now rarely used in clinics. The lack of understanding of meningeal mechanics data makes it difficult to develop instruments that are capable of accurately capturing these characteristics. CSF flow dynamics are regulated by PBMs, which achieve this by promoting the accumulation of ECM proteins and impeding CSF entry into perivascular spaces [3]. The study of mechanical properties in different regions can also help us infer the distribution of immune cells [63] and lay a foundation for further disease specific therapeutic treatment.

So far, many studies have experimentally characterized the regional difference of dura meninges via macroscopic elastic, shear modulus and uniaxial tensile loading. Significant regional differences in mechanical stiffness were observed, irrespective of directionality. It may be attributed to the increased collagen I and elastin association contributing to higher stiffness. However, more in-depth studies of the influence of dura meninges ECM components on its biomechanics should be considered in future investigations. In contrast to the dura meninges, few studies have reported about PAC, especially its biomechanics and regional difference. For a comparative analysis of regional differences in PAC, mechanics are presented in Table 1.

**Table 1.** Summary of reported experimental regional differences in PAC mechanical properties

Tissue type	Test type	Macro or micro	Test regions	Regional difference	Causes associated with differences	Refs.
Porcine cranial PAC	Shear model and AFM indentation	Macro and micro	Temporal, frontal, parietal, occipital piriform cerebellum lobes	Cerebellum lobe was higher than other lobes	Thickness, collagen I, elastin, and other supported proteins	This study
Rat cranial PAC	Microindentation	Micro	Cerebellar and cortical PAC	No significant difference	Thickness, collagen I, and vimentin	[29]
Ovine cranial PAC	Uniaxial tension	Macro	Occipital and frontal lobes, for left and right hemispheres	No significant difference	Biomechanical properties	[24]
Bovine cranial PAC	Shear loading	Macro	Temporal, frontal, parietal, and occipital lobes	No significant difference	Different strain rates	[23,63]

At the beginning, the PAC has been identified as a bulk homogeneous, isotropic structure. With the further development of research, the bulk tissue-level studies exhibited isotropic behavior. However, Jin et al. [23,64] found a clear anisotropy in the mechanical response of the PAC via uniaxial tensile loading experiments at different strain rates. Subsequent observations in rat and ovine PAC further supported this trend. Although the evidence is not conclusive and significant yet, there is a consensus that PAC has some regional mechanical differences.

In our study, rather than observing the accurate macroscopic and microscopic tissue properties, we focused on analyzing the regional differences of the mechanical responses of the PAC and their underlying mechanisms. It is widely recognized that the mammalian PAC exhibits complex regional variability, influenced by the density of arachnoid trabeculae and the distribution of subarachnoid vasculature [65]. Upon removal from the body, the meninges lose the supporting CSF, causing immediate collapse and complicating mechanical tests. The preparation of PAC samples for testing involves surmounting numerous substantial technical hurdles: firstly, a detailed protocol to obtain and preserve fresh PAC; secondly, anatomically subdividing the meninges according to brain regions; thirdly, isolating the PAC layers and fixing them under the test instrument while maintaining hydration and being immutable during the test.

Moreover, due to the PAC’s extreme fragility, traditional uniaxial tensile loading experiments are not suitable. As previously reported in the literature, such methods did not yield significant differences [23,24,64]. In order to further report the differences in their mechanical properties, rheometers and shear models were applied. Parkins et al. [35] first reported the rheological properties of human brain tissue; this method can explore the adaptation of hydrogel microarray to tumor tissue on a macro level. The primary objective of our study was to characterize regional rheological heterogeneity in the PAC. In this experiment, we observed that the mechanical properties of cerebellar regions are significantly higher than those of other regions, which may be partially due to the thickness of cerebellar regions. Since the thickness of bovine PAC is lower than that of porcine PAC and the measurement instruments used are different, the experimental data remained within the same order of magnitude, indicating the accuracy of the data [23]. We postulate that the greater anatomical scale of the porcine brain may lead to more pronounced regional variations in physiological stresses and CSF dynamics, contributing to the observed differences in mechanical properties.

In order to further verify the conjecture of the macroscopic experiment, we conducted micromechanical testing using AFM. This approach, combined with the mode of using measurements under the fluid, may explain the lower elastic modulus we observe compared to the shear model. Notably, according to the AFM measurements of PAC in mice, our micromechanical properties are consistent with the observed values within the same order of magnitude [29]. In mouse models, the authors reported that the PAC thickness in cerebellar regions was significantly higher than in other regions, yet no statistical differences in mechanical properties were detected. The reason may be that the PAC of mice is too thin, and the differences are not obvious. In this experiment, we amplified these differences through the leverage of a porcine model, enabling us to detect elevated mechanical properties in the cerebellar region at the micro level. However, because the mechanical properties of the vascular region are so different from other regions, we could not test it in liquid mode. In our experiments, we did not use fresh immunofluorescence staining, as AFM microscopes are not equipped with fluorescence capabilities, preventing simultaneous mechanical and fluorescent imaging.

Notably, prior literature has demonstrated that the salt ions in the tissue preservation solution, and immobilization under the tissue in AFM measurements, do not affect the mechanical properties of the tissue. The most noteworthy variable is the influence of time. According to previous literature reports, 48 h is often cited as an optimal testing window. In order to be more accurate, we take no more than 24 h from fresh sample to test.

To further confirm the differences in mechanical properties, we investigated the role of supporting proteins such as collagen I and elastin, which may explain the elevated mechanical properties in the cerebellar regions [66,67]. We chose the 2-photon microscope for the following reasons: first, it is more suitable for in vivo detection; second, it can directly display the distribution of supporting proteins; third, it does not need staining and can be directly observed. Because uniform staining is difficult in very thick samples, 2-photon microscopy overcomes this problem. A potential explanation for the observed difference is that a nonuniform spatial distribution of cells within the region led to an apparent increase in the expression of certain genes.

Our findings are also in line with previous studies on the difference in the mechanical properties of the dural region. According to previous reports, AD patients exhibit altered CSF flow dynamics, which may upgrade the expression of collagen I and ECM protein, leading to PAC thickening. Previous studies have shown that CSF flows from the fourth ventricle into the SAS via 3 critical openings: the median aperture and the paired lateral apertures. The median aperture directs CSF posteriorly into the cerebellomedullary cistern, while the lateral apertures allow CSF to circulate anteriorly around the brainstem into the pontine cistern. From these entry points, CSF propagates throughout the SAS, enveloping the entire brain (including the cerebral hemispheres and cerebellum) and spinal cord. The CSF circulation further confirmed the mechanical properties of the cerebellar PAC region over other regions. Furthermore, we postulate that the greater anatomical scale of the porcine brain may lead to more pronounced regional variations in physiological stresses and CSF dynamics, contributing to the observed differences in mechanical properties.

A notable limitation of our study is that the PAC tissue loses its physiological pressurization immediately upon being peeled from the brain, which may substantially alter its mechanical response. Immersion in preservative solutions may also induce tissue swelling or shrinkage, as these conditions do not replicate the natural osmotic environment of the meningeal tissue. In addition, PAC is a 2-layer membrane structure, and the mechanical properties of the 2 membranes, as well as the mechanical properties of the blood vessels sandwiched between them, are inconsistent. The loss of intermediate fluid makes it difficult to distinguish the specific source of the difference. On the other hand, whether in vivo or in vitro, it is almost impossible to separate the 2 membranes. Thus, advancing from the current situation requires a significant improvement for in vivo analysis, such as invasive approaches. Successfully pursuing these paths will help us obtain the accurate, functionally relevant data, for enhancing the predictive tools like FE models and to deepening our understanding of CSF monitors and meningeal function. In addition, accurate measurement of physiological data can better predict respiratory motion during surgery, thereby enhancing the precision and safety of robot-assisted procedures while also improving the accuracy of interaction recognition [68].

In addition, as previously reported, fresh thick tissue samples are really challenging to stain uniformly. This limitation hinders our methods from exploring the reasons for the differences on a deeper scale. Furthermore, our AFM is not equipped with other microscopes, so it is not possible to explore the relationship between mechanical property differences and protein expression simultaneously. At the same time, due to the lack of disease samples, we cannot compare the regional dynamics between the normal and the disease groups. Comparative studies using relevant disease models (e.g., AD transgenic pigs or mice) will help elucidate the role of PAC mechanics in disease progression.

A study has demonstrated that predictions of extra-axial hemorrhage after TBI will be enhanced by accounting for the PAC’s regional variations [25]. In the future, if we can collect relevant data indicators of human’s PAC, it will make a great contribution to the prediction of the FE model. Next, if we can inject gel or liquid into the tissue before it leaves the body, enabling complete isolation of the PAC and acquisition of true physiological mechanical data, it will accelerate our understanding of the mechanical properties of the PAC. In addition, look forward to future collaborations with computational modeling methods, like FE models, to more accurately predict stress–strain distributions in the brain during TBI events. In the future, integrating our testing results with techniques such as polarized light microscopy, SALS images will enable a more comprehensive, multiscale characterization of PAC’s anisotropic mechanics. Additionally, if we can explore the differences in immunological properties caused by mechanical properties, it will have a great impact on our local drug delivery and clinical intervention. Furthermore, an understanding of the mechanical properties of the leptomeninges provides a theoretical foundation for the development of novel brain–computer interface materials [69] and wearable brain devices [70].

## Conclusion

The objective of our work is to explore the differences in regional mechanical properties and the possible biochemical reasons for these differences via macro biomechanics (rheology) and micro biomechanics (AFM indentation test), 2-photon microscope techniques. For the first time, we characterize regional mechanical differences in the porcine PAC, which has the most identical brain structure to humans after primates. Our results suggest that the values of many mechanical properties of cerebellar regions were higher than those of other regions at the macro and micro levels, which may be related to the direction of CSF flow and the distribution of supporting proteins.

## Data Availability

The data are contained within the article.
